# Characterization of alterations in spontaneous behaviors in a rat model of neuropathic pain - new outcome measures for pain evaluation?

**DOI:** 10.3389/fnbeh.2025.1550476

**Published:** 2025-06-06

**Authors:** Annamária Liptáková, M. J. Castelhano-Carlos, Juliana Fiúza-Fernandes, Nuno Sousa, Michelle Roche, David P. Finn, Hugo Leite-Almeida

**Affiliations:** ^1^Life and Health Sciences Research Institute (ICVS), School of Medicine, University of Minho, Braga, Portugal; ^2^ICVS/3B’s - PT Government Associate Laboratory, Braga/Guimarães, Portugal; ^3^Pharmacology and Therapeutics, School of Medicine, University of Galway, Galway, Ireland; ^4^Galway Neuroscience Centre, University of Galway, Galway, Ireland; ^5^Centre for Pain Research, University of Galway, Galway, Ireland; ^6^Centro Universitário da Jaguariúna (UniFAJ), São Paulo, Brazil; ^7^Centro Universitário Max-Planck (UniMAX), São Paulo, Brazil; ^8^Physiology, School of Medicine, University of Galway, Galway, Ireland

**Keywords:** chronic pain, PhenoWorld, home-cage behavior, spontaneous behavior, ethology, sex differences, light-dark cycle

## Abstract

Chronic pain affects all life domains including social interaction and responding. The aim of this study was to track spontaneous behaviors in an experimental chronic pain model to uncover alternative pain indicators in a socially and physically enriched home-cage setting. The spared nerve injury (SNI) was used to model neuropathic pain in Wistar Han male and female rats housed in the PhenoWorld (PhW). Spontaneous behavior of animals was recorded in their home cages once a week following SNI during both the dark and light phases of the light-dark cycle using focal sampling in order to assess alterations induced by neuropathic pain. Males and females with SNI demonstrated significantly lower threshold to von Frey test (VF) in the ipsilateral hind paws compared to sham controls. SNI significantly increased huddling time in both males and females during light and dark phases. Males showed increased grooming and play fighting during the dark phase compared to females while during the light phase females huddled significantly more than males. No significant effects were observed on other behaviors analyzed. This study showed that peripheral nerve injury has an impact on spontaneous behavior, specifically on huddling. This finding provides new perspective into pain evaluation and suggests the possibility of considering spontaneous behavior as an additional method of assessing pain-related behavior in rodents. To bridge the current gap between basic research and development of novel analgesics, there is a need to develop non-evoked behavioral assays to investigate changes in animal wellbeing and spontaneous pain. Our findings raise the possibility of discovering new outcome measures, however, additional study to reverse these behaviors with analgesics should be conducted.

## 1 Introduction

Chronic pain represents a major public health problem impacting over 30% of individuals globally (Cohen et al., 2021). It has a complex pathophysiology and affects patients’ emotional state as well as sociability (Cohen et al., 2021; Domenichiello and Ramsden, 2019). Currently available pain therapies have adverse side effects and limited efficacy which makes the search for novel analgesics highly important (González-Cano et al., 2020). However, there is an evident gap between basic research and drug development, with only 11% of investigated analgesics in clinical trials eventually approved (Mao, 2012; Hay et al., 2014).

Multiple factors hinder effective translation of candidate analgesics to the clinic. For instance, a cardinal feature of chronic pain in human patients is spontaneous pain, however this is hard to capture (and frequently ignored) in animal models of chronic pain. However, some labs have been attempting to examine affective and spontaneous pain behavior using a variety of approaches, particularly those that have an emotional and cognitive dimension (Guerreiro et al., 2022; Guimarães et al., 2019; Guimarães et al., 2021; see for reviews: Cunha et al., 2020b; Leite-Almeida et al., 2015). Other behavioral manifestations like weight-bearing, gait analysis, burrowing, nesting, voluntary wheel running, ultrasound vocalization, facial grimace scale (Urban et al., 2011; Tappe-Theodor and Kuner, 2014; Hasriadi et al., 2021; Zentrich et al., 2021; Burand et al., 2023) and machine learning approaches for behavioral phenotyping (Jones et al., 2020; Sabnis et al., 2022; Burdge et al., 2024) have also been adopted in the context of preclinical chronic pain studies to provide non-evoked measures of pain-related behavior. In addition, pain behavior can be evaluated with almost no human intervention by home-cage monitoring systems resulting in more naturalistic, long-term observations of spontaneous behaviors (Grieco et al., 2021; Zentrich et al., 2021). Various automated home-cage monitoring systems have been developed, including the PhenoWorld (PhW) consisting of an automated testing apparatus where animals can be group-housed while home-cage activity is being monitored (Amorim et al., 2022; Castelhano-Carlos et al., 2014; see for review: Leite-Almeida et al., 2022).

Herein, we monitored spontaneous behaviors of rats in their home-cage and assessed pain-induced alterations using the spared nerve injury neuropathic pain model (SNI; Decosterd and Woolf, 2000), with a focus on sex differences. The aim of this study was to uncover alternative pain-related indicators in rats by screening behavior during both light and dark phases, over a four-week period post-SNI, while being undisturbed in their home-cage. As a housing environment, we used the PhW, a validated paradigm for screening rodent behavior, where animals lived in a group of six, with an opportunity to perform natural behaviors (Castelhano-Carlos et al., 2014).

## 2 Materials and methods

### 2.1 Animals and housing conditions

Thirty-six Wistar Han male and female rats, 2–5 months old, were bred in our animal facility. All animals were maintained under standard laboratory conditions with reversed 12 h light/ 12 h dark cycle (lights on from 8:00 p.m. to 8.00 a.m.), 21 ± 1°C ambient temperature and 50%–60% relative humidity. Animals were provided with a standard diet (4RF21; Mucedola S.R.L., Italy) and autoclaved water *ad libitum*.

Animals were housed in the PhW, (TSE Systems GmbH, Germany) – a paradigm representing a semi-natural rodent housing environment. The PhW consists of a 1 m^2^ and 50 cm height central cage covered with a corncob bedding (Scobis Due; Mucedola SRL, Settimo Milanese, Milan, Italy), connected to a box with four running wheels by means of two open access tubes and to two drinking/feeding boxes. All PhW areas were covered by either perforated Plexiglas or stainless-steel grids. The central cage was enriched with one plastic and two cardboard tubes. Animals lived in the PhW in groups of six rats with free access to all the connected boxes.

One week prior, animals were acclimatized to inverted light/dark cycle in standard filter-topped transparent type III cages (ref. 1291H, Tecniplast; Italy) with pair-housed animals (STD2 cages) in the room with PhW paradigm. Afterwards they were moved to the PhW, for a week of acclimatization to a new housing environment before spared nerve injury surgery was performed.

Animals were handled by the same experimenter throughout the study who performed all procedures including the maintenance and cleaning of the PhW every 2 weeks. During bedding exchange and cleaning of PhW’s compartments animals were housed in a standard cage for six rats (ref. 2000P, Tecniplast, Italy).

The experiment was performed in six different cohorts because of the limitation in number of animals housed in the PhW. Each cohort included six rats, except the first cohort, which involved five rats because of the death of one animal after spared nerve injury surgery. Cohorts one and five represent male SNI group (total *n* = 11), cohorts two and six represent female SNI group (total *n* = 12), cohort three corresponds to male sham group (*n* = 6) and cohort four corresponds to female sham group (*n* = 6). The cohorts were run in the order one to six sequentially. This experiment was conducted as part of a larger study where Light/Dark Box test and Forced Swim test were also performed prior SNI/Sham surgery as baseline measures. However, the present paper focuses on the home-cage monitoring of spontaneous behaviors, while the data related to anxiety- and depression-like behaviors will be presented in a subsequent publication.

All procedures involving animals were approved by the respective local organizations and experiments were performed according to the European Directive 2010/63/EU.

### 2.2 Neuropathic pain model: spared nerve injury

The spared nerve injury (SNI) model was used to model neuropathic pain (Decosterd and Woolf, 2000). Animals were deeply anesthetized with 1.5:1 mixture of 75 mg/Kg of ketamine (Imalgene^®^, 100 mg/mL – Merial, Lyon, France) and 1 mg/Kg of medetomidine (Dormitor^®^, 1 mg/mL – Orion Pharma, Espoo, Finland) (Esteves et al., 2019). The SNI model was performed as previously described (Cunha et al., 2020a). Briefly, it comprised of a ligation and a distal transection of a common peroneal and tibial branch of the right sciatic nerve while leaving the sural branch intact. Sham controls surgery involved an exposure of the sciatic nerve and its branches without any lesion. After surgery, anesthesia was reversed with the administration of 1mg/kg of atipamezole (Tipafar^®^, 5 mg/ml – VetPharma Animal Health, Barcelona, Spain) subcutaneously. Animals were left to recover from anesthesia in STD2 cages in the recovery room under the red lamp until being fully awake. Afterward they were transported to the PhW. Seven days after surgery we started to collect behavioral data as explained below.

### 2.3 Assessment of mechanical hypersensitivity

Mechanical hypersensitivity was measured based on the up-and-down method using von Frey (VF) monofilaments (Chaplan et al., 1994). Briefly, animals were placed in an elevated grid and left to habituate for 5 min. Afterward a series of calibrated VF monofilaments: 15.0, 8.0, 6.0, 4.0, 2.0, 1.0, 0.6 and 0.4 g (North Coast Medical Inc.) starting with 2.0 g monofilament was applied to the lateral plantar surface of the right hind-paw. The test would advance upward, if no response was elicited (= 0), or downward, if a brisk withdrawal of the limb was produced (= X), until six applications were obtained around the threshold point according to the model developed by Dixon (Dixon, 1980). The 50% threshold was calculated using the following formula: 50% threshold (g) = 10^*Xf*+*kδ*^/10,000, where Xf is the value (in log units) of the final VF filament; K is the tabular value corresponding to pattern of positive/negative responses (X and 0 sequence) and δ is the average difference (in log units) between VF hairs used (Guimarães et al., 2019).

### 2.4 Focal sampling home-cage behavior

**FIGURE 1:**
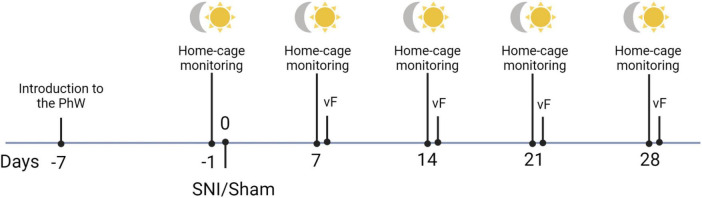
Schematic timeline of the experimental procedures.

Focal sampling analysis at the home-cage was used to assess behaviors performed in the home cage under SNI and sham conditions in both sexes. Animals were video recorded once a week (in both light and dark phases of the light-dark cycle) starting from a baseline week (1 week after introduction to the PhW) until the fourth week after SNI or sham surgery (7, 14, 21 and 28 days after surgery; Figure 1). Recordings were made in the middle of a dark phase (2–4 p.m.) and in the middle of a light phase (2–4 a.m.); the tail of each animal was marked 2 days before the recording day with a pen, to avoid any disturbance of behavior and allow identification of the animals. Sampling of the videos was made as follows: 2 h records were divided into seven videos of 17 min, from each of these videos a sample from the minute 8 until minute 13 was manually scored. The time spent performing various patterns of behavior for each of the 5 min time bins over a 2 h period was recorded and analyzed. The observed behaviors were classified as exploratory behavior, self-grooming, sleep/rest alone, social interaction, huddling, a play fight, following/chasing and central cage absence. The classification of behaviors resulted from pilot observation. For the description of patterns of behaviors scored see Table 1.

**TABLE 1:** Ethogram of home-cage behaviors.

Behavior	Description
1. Exploring	Included rearing, sniffing, interaction with objects, walking, jumping and scanning.
2. Self-grooming	It is a self-maintenance behavior, performed by licking all reachable parts of the body and nibbling its fur.
3. Sleep/rest alone	Scored when a rat slept or rested (not moving) alone.
4. Social behavior	
• Social interaction	Included allogrooming, approaching, sniffing the partner and interaction with other cage mate without any signs of wrestling and self-defence.
• Huddling	Two or more animals sleeping/resting in a proximate contact with each other.
• Fighting (a play fight)	Involved attack and defence of the nape of the neck, wrestling, pinning.
• Following/chasing	As following/chasing we scored when both animals were in motion, one chasing another while moving within a tail length distance.
5. Central cage absence	Scoring time when animals were not present in the central cage provided us with an information about place preference between the PhW compartments.

### 2.5 Statistical analysis

All statistical analysis was conducted using JASP software (version 0.16.2). Normality and homogeneity of variance were evaluated using Shapiro-Wilk test and Bartlett’s test, respectively. Normally distributed data was analyzed using two-way ANOVA followed by Tukey *post-hoc* test to compare home-cage behaviors between surgery groups and sexes. Two-way repeated measures (RM) ANOVA was conducted to compare different time points in chronic pain development. The assumption of sphericity was tested using Mauchly’s test and in cases where the assumption of sphericity was violated, Greenhause-Geisser correction was applied to adjust the degrees of freedom. Pearson correlation coefficients were calculated to investigate the relationship between pain perception and home-cage behaviors. If data failed to meet the assumptions of normality and/or homogeneity of variance, three transformations were applied sequentially to assess whether parametric statistics could be performed: square root of the data values, log of the data values, and ranking of the data values. If the distribution and/or homogeneity of the data set was not corrected after any of these transformations, and the highest standard deviation was two times higher than the smallest standard deviation of the data set being analyzed, non-parametric Kruskal-Wallis test followed by a *post hoc* Mann-Whitney U test (*p*-value adjusted with Bonferroni-Holm corrections for multiple comparisons) was used to analyze the data. Differences were considered to be statistically significant if *p* < 0.05. Graphs were constructed using Graphpad Prism and data presented are expressed as group mean ± S.E.M for parametric data and as median with interquartile range (IQR) for non-parametric data. All outputs of the statistical analysis are presented in Supplementary Tables 1–7.

## 3 Results

### 3.1 Mechanical hypersensitivity

**FIGURE 2:**
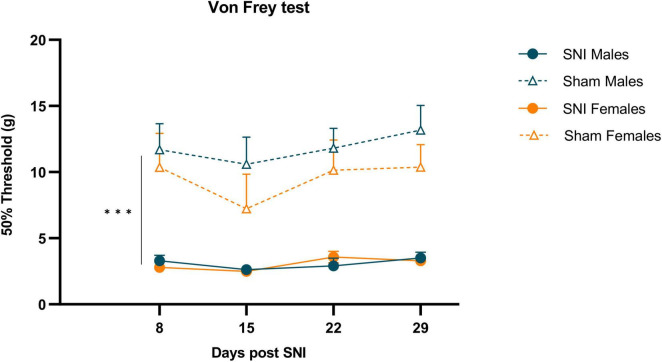
Mechanical hypersensitivity induced by SNI in both sexes. Data are expressed as mean ± S.E.M. *N* = 12/SNI group, *n* = 6/sham group. RM two-way ANOVA. ***p < 0.001 SNI vs Sham.

Male and female rats developed mechanical hypersensitivity after SNI that persisted at least 29 days, which was demonstrated by a significantly lower threshold to VF in the ipsilateral hind paws of SNI rats compared to sham controls (Two-way RM ANOVA: F_(1,30)_ = 93.888, *p* < 0.001, η_*p*_^2^ = 0.758; Figure 2). There were no differences in withdrawal thresholds between male and female rats after SNI or sham surgery.

### 3.2 Effects of spared nerve injury and sex on spontaneous home-cage behaviors in the dark phase

**FIGURE 3:**
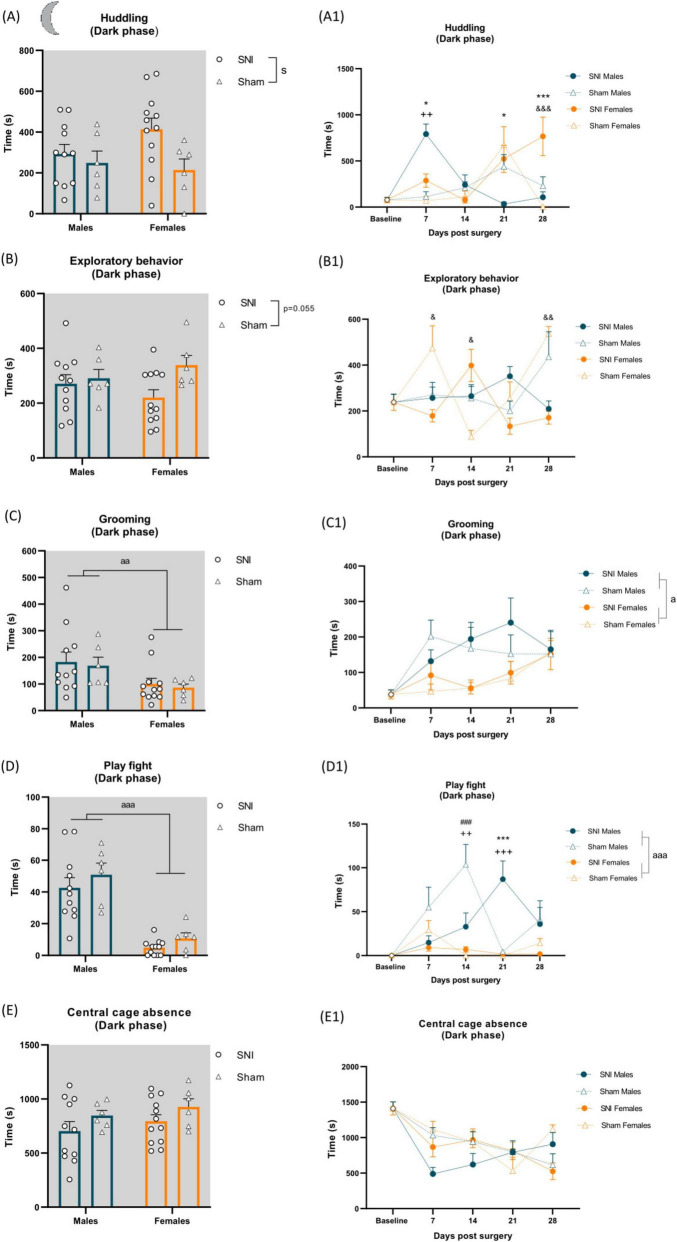
Left panel shows alterations of spontaneous behavior induced by SNI in the dark phase of the cycle and sex differences. Data represent average of 4 weeks behavior performed after SNI/sham surgery **(A–E)**. Data are expressed as mean ± S.E.M. *N* = 12/SNI group, *n* = 6/sham group. Two-way ANOVA followed by Tukey *post hoc* test. Main effect of surgery: *^s^p* < 0.05 SNI vs sham; Main effect of sex: *^aa^p* < 0.01, *^aaa^p* < 0.001 Male vs Female. Right panel shows changes in behavior over time during the dark phase. RM two-way ANOVA followed by Tukey *post hoc* test. Main effect of sex: *^a^p* < 0.05, *^aaa^p* < 0.001. Time × surgery × sex interaction: ++*p* < 0.01, +++*p* < 0.001 indicating effect of SNI in males at particular time point, &*p* < 0.05, &&*p* < 0.01, &&&*p* < 0.001 indicating effect of SNI in females at particular time point, *p < 0.05, ***p < 0.001 indicating effect of sex in SNI rats, ###*p* < 0.001 indicating effect of sex in sham rats **(A1–E1)**.

Measurements of home-cage behavior were performed before SNI/sham surgery as a baseline measure and on post-operative days (POD) 7, 14, 21 and 28. A two-way ANOVA revealed a significant main effect of injury on huddling behavior during the dark phase of the light/dark cycle, with SNI rats spending more time huddling compared to sham animals (F_(1,31)_ = 4.406, *p* = 0.044, η_*p*_^2^ = 0.124; Figure 3A and Supplementary Table 1). A two-way RM ANOVA further revealed a significant time × surgery × sex interaction [F_(2.569,79.645)_ = 4.793, *p* = 0.006, η_*p*_^2^ = 0.134; Supplementary Table 5]. Tukey’s *post-hoc* tests indicated that SNI males spent significantly more time huddling on POD 7 compared to sham males (*p* = 0.006), while SNI females exhibited significantly increased huddling on POD 28 compared to sham females (*p* < 0.001). Additionally, *post-hoc* analysis revealed a significant sex difference within the SNI group, with males spending more time huddling than females on POD 7 (*p* = 0.025). However, on POD 21 (*p* = 0.035) and POD 28 (*p* < 0.001), huddling behavior was significantly reduced in SNI males compared to SNI females (Figure 3A1 and Supplementary Table 6).

Exploratory behavior was not significantly altered by the injury when averaged over 4 weeks post-SNI/sham surgery [F_(1,31)_ = 3.967, *p* = 0.055, η_*p*_^2^ = 0.113; Figure 3B and Supplementary Table 1]. However, a two-way RM ANOVA revealed a significant time × surgery × sex interaction [F_(4,124)_ = 11.007, *p* < 0.001, η_*p*_^2^ = 0.262; Supplementary Table 5) with further Tukey’s *post-hoc* analysis indicating reduced exploratory behavior in SNI females on POD 7 (*p* = 0.041) and POD 28 (*p* = 0.002) compared to their sham counterparts, whereas on POD 14 (*p* = 0.023), exploratory behavior was increased in SNI females relative to their sham counterparts (Figure 3B1 and Supplementary Table 6).

A two-way ANOVA revealed a significant main effect of sex on grooming [F_(1,31)_ = 8.190, *p* = 0.007, η_*p*_^2^ = 0.209; Figure 3C and Supplementary Table 1] regardless of surgery, with males spending more time grooming than females, which was further supported by a two-way RM ANOVA over the 4 weeks time course, which also indicated a main effect of sex [F_(1,31)_ = 6.145, *p* = 0.019, η_*p*_^2^ = 0.165; Figure 3C1].

Males spent significantly more time play fighting compared to females [F_(1,31)_ = 54.219, *p* < 0.001, η_*p*_^2^ = 0.636; Figure 3D and Supplementary Table 1], regardless of surgery. A two-way RM ANOVA confirmed a main effect of sex [F_(1,31)_ = 54.219, *p* < 0.001, η_*p*_^2^ = 0.636], indicating increased play fighting in males. Additionally, a significant time × surgery × sex interaction was observed [F_(4,124)_ = 5.198, *p* < 0.001, η_*p*_^2^ = 0.144; Supplementary Table 5]. *Post hoc* analysis revealed increased play fighting in sham males on POD 14 (*p* < 0.001) and in SNI males on POD 21 (*p* < 0.001) compared to their respective female counterparts. Furthermore, SNI reduced play fighting in males on POD 14 (*p* = 0.008) but increased it on POD 21 (*p* < 0.001) relative to their sham male counterparts (Figure 3D1 and Supplementary Table 6). Following/chasing was a rare behavior observed during the dark phase. A Kruskal-Wallis test followed by a Mann-Whitney U *post hoc* test revealed a significant increase in following/chasing in SNI males compared to SNI females [H(3) = 12.84, *p* = 0.005; U = 15, *p* = 0.0005, Supplementary Table 2), further supported by a two-way RM ANOVA, which indicated a significant time × surgery × sex interaction [F_(4,124)_ = 2.837, *p* = 0.027, η_*p*_^2^ = 0.084; Supplementary Table 6). *Post hoc* analysis confirmed a significant increase in following/chasing in SNI males on POD 21 compared to SNI females (*p* < 0.001) (not shown, Supplementary Table 6).

No differences were observed in central cage absence (Figures 3E, E1 and Supplementary Table 1), sleep/rest alone (not shown; Supplementary Table 3) and social interaction (not shown; Supplementary Table 1).

To investigate the potential relationship between mechanical hypersensitivity and spontaneous behaviors we carried out correlation analysis of VF thresholds along the 4 weeks averages with the averages of the 4 weeks 5 min time bins over a 2 h time spent performing behaviors, using Pearson correlation. The correlation analysis revealed a significant positive correlation between exploratory behavior and VF thresholds (r = 0.6507, *p* = 0.0301) and fighting and VF thresholds (r = 0.6607, *p* = 0.0269) in males, during the dark phase (Figure 5B). VF thresholds were not significantly correlated with any of the other scored behaviors (Figure 5A). There was no significant correlation between any behavior and VF thresholds in females.

### 3.3 Effects of spared nerve injury and sex on spontaneous home-cage behaviors in the light phase

**FIGURE 4:**
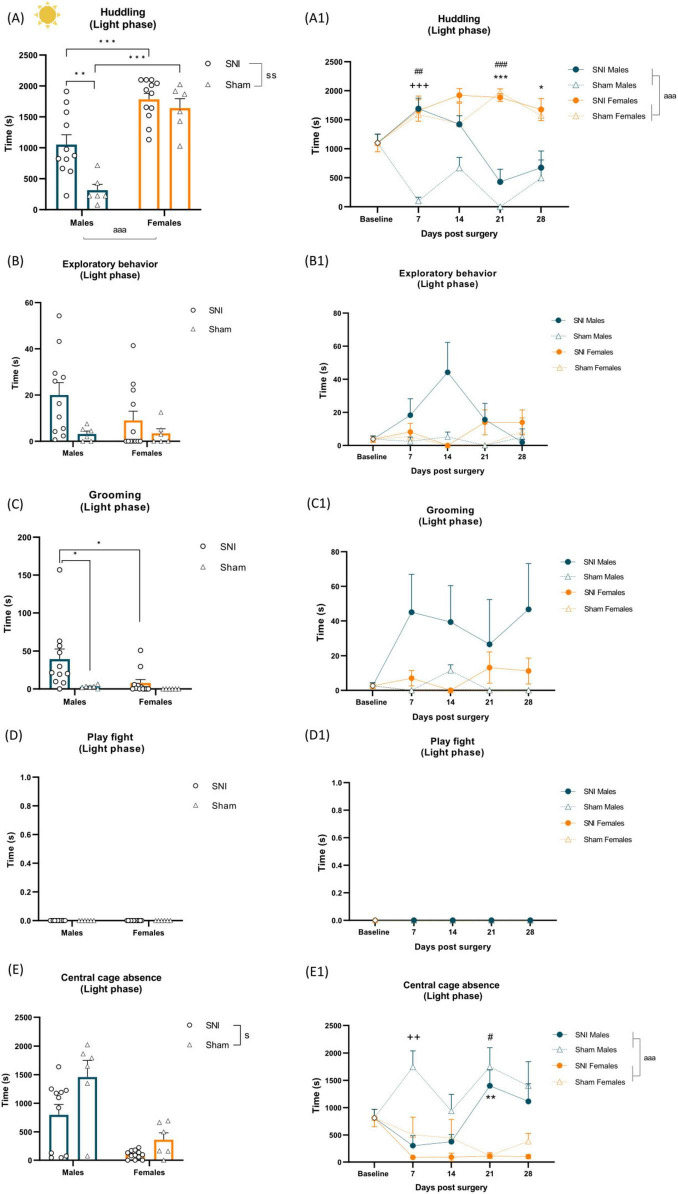
Left panel shows alterations of spontaneous behavior induced by SNI in the light phase of the cycle and sex differences. Data represent average of 4 weeks behavior performed after SNI/sham surgery **(A–E)**. Data are expressed as mean ± S.E.M/median with IQR. *N* = 12/SNI group, *n* = 6/sham group. Two-way ANOVA followed by Tukey *post hoc* test **(A, B, D, E)**/Kruskal-Wallis with Mann-Whitney U *post hoc* test **(C)**. Main effect of surgery: *^s^p* < 0.05, *^ss^p* < 0.01 SNI vs sham. Main effect of sex: *^aaa^p* < 0.001 Male vs Female. Surgery × sex interaction: *p < 0.05, **p < 0.01, ***p < 0.001. Right panel shows changes in behavior over time during the light phase. RM two-way ANOVA followed by Tukey *post hoc* test. Main effect of sex: *^aaa^p* < 0.001. Time × surgery × sex interaction: ++*p* < 0.01, +++*p* < 0.001 indicating effect of SNI in males at particular time point, **p < 0.01, ***p < 0.001 indicating effect of sex in SNI rats, #*p* < 0.05, ##*p* < 0.01, ###*p* < 0.001 indicating effect of sex in sham rats **(A1–E1)**.

Two-way ANOVA revealed significant main effects of SNI surgery [F_(1,31)_ = 9.623, *p* = 0.004, η_*p*_^2^ = 0.237], sex [F_(1,31)_ = 52.506, *p* < 0.001, η_*p*_^2^ = 0.629) and a surgery × sex interaction [F_(1,31)_ = 4.399, *p* = 0.044, η_*p*_^2^ = 0.124) on huddling behavior during the light phase of the light/dark cycle. SNI surgery increased huddling time compared to sham controls, and females exhibited greater huddling duration than males. *Post hoc* analysis showed that SNI males exhibited increased huddling compared to sham males (*p* = 0.005), while both SNI (*p* < 0.001) and sham females (*p* < 0.001) huddled more than their respective male counterparts (Figure 4A and Supplementary Table 3). Two-way RM ANOVA further revealed a significant main effect of sex [F_(1,31)_ = 44.538, *p* < 0.001, η_*p*_^2^ = 0.590), and a time × surgery × sex interaction [F_(4,124)_ = 4.979, *p* < 0.001, η_*p*_^2^ = 0.138, Supplementary Table 5]. *Post hoc* analysis indicated increased huddling in SNI males on POD 7 compared to sham controls (*p* < 0.001). Additionally, increased huddling was indicated in sham females on POD 7 (*p* = 0.01) and POD 21 (*p* < 0.001) compared to sham males, while SNI females spent more time huddling on POD 21 (*p* < 0.001) and POD 28 (*p* = 0.026) compared to SNI males (Figure 4A1 and Supplementary Table 6).

**FIGURE 5:**
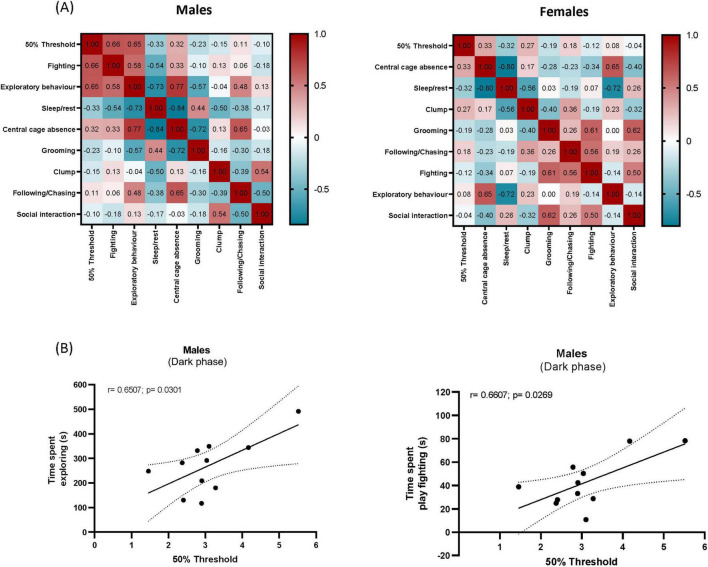
Correlation matrices. Data represent the average behavioral and pain threshold measurements recorded over four weeks following SNI surgery during the dark phase. Pearson correlations between measured behavioral outputs are presented for males and females **(A)**. Exploratory behavior and play fighting exhibited a positive association with paw withdrawal thresholds (g) in SNI males **(B)**, suggesting that these behaviors might be more susceptible to the impact of pain.

Kruskal-Wallis with Mann-Whitney U *post hoc* test revealed a significant increase in grooming in SNI males compared to both sham males [H(3) = 17.48, *p* = 0.0006; U = 5.5, *p* = 0.0032; Figure 4C and Supplementary Table 4] and SNI females during the light phase [H(3) = 17.48, *p* = 0.0006; U = 21, *p* = 0.004; Figure 4C and Supplementary Table 4]; see also Figure 4C1 for a longitudinal outlook. Furthermore, two-way ANOVA showed a significant decrease in central cage absence following SNI surgery [F_(1,31)_ = 6.460, *p* = 0.016, η_*p*_^2^ = 0.172; Figure 4E and Supplementary Table 3]. RM ANOVA revealed a main effect of sex [F_(1,31)_ = 23.233, p < 0.001, η_*p*_^2^ = 0.428], with males increased central cage absence compared to females, as well as time × surgery × sex interaction [F_(4,124)_ = 3.548, *p* = 0.009, η_*p*_^2^ = 0.103, Supplementary Table 5). *Post hoc* analyses indicated that SNI surgery reduced central cage absence in males on POD 7 compared to sham males (*p* = 0.010), while on POD 21, both SNI (*p* = 0.003) and sham males (*p* = 0.011) exhibited greater central cage absence than their female counterparts (Figure 4E1 and Supplementary Table 6).

No differences were observed in exploratory behavior (Figures 4B, B1 and Supplementary Table 4) and sleep/rest alone during the light phase (Supplementary Table 4). Social interaction, play fight (Figures 4D, D1) and following/chasing were not observed during the light phase of the light/dark cycle.

## 4 Discussion

Chronic pain is, by definition, persistent or recurrent and is frequently of a spontaneous nature (Hylands-White et al., 2017). While it can be experienced at any time, most preclinical rodent studies are performed during the light period, the natural rodent resting period (Pitzer et al., 2016; Hasriadi et al., 2021; Amorim et al., 2022). Also, information is collected in small time windows, typically under conditions that require some manipulations and/or restraint, which may induce stress-induced analgesia or hyperalgesia (Pitzer et al., 2016). We therefore performed a study to screen spontaneous home-cage behavior during dark and light phases to detect potential alterations in a rat model of neuropathic pain in both sexes. This approach has several potential advantages, namely reducing excessive manipulation of the animals and taking advantage of animals’ natural behavior. Given that this study was conducted in an environmentally enriched setting, assessing home-cage behavior in standard housing might yield different outcomes. Moreover, it is important to keep in mind that interpreting longitudinal data requires careful consideration, as certain variations may represent extreme values recorded within the specific time windows. While the underlying causes are not always clear (e.g., pain fluctuations, noises in the facilities, odors), they do not appear to impact the overall trends as presented in the four-week average of the aggregated data.

Focal sampling of animals living in the PhW’s home-cage revealed a significant effect of SNI surgery on huddling and exploring during the dark phase and on huddling and grooming behavior during the light phase of the cycle. While SNI leads to a decreased exploration during the dark phase, in the light phase we observed the opposite effect. Exploratory behavior included a range of behaviors, such as rearing, sniffing, interaction with objects, walking, jumping and scanning. Decrease in exploratory behavior is in line with previous studies that assessed the effects of either neuropathic or inflammatory pain on locomotion (Bree et al., 2016; Alsalem et al., 2020; Hasriadi et al., 2021). However, it is important to note that different results have also been reported, showing no pain-related effects on locomotor behavior (Sheahan et al., 2017; Burek et al., 2021). Increased exploration and grooming during the light phase, inactive period during which rodents usually rest, might be associated with pain-induced sleep disturbances. These are often reported in chronic pain patients (Duo et al., 2023; Tang, 2008), although our results did not show any effect of SNI on sleep/rest behavior, as observed by video-assisted focal sampling. Similarly, SNI and Complete Freund’s adjuvant (CFA) did not alter time spent inactive while Chronic constriction injury (CCI) model of neuropathic pain increased inactivity time during the initial monitoring days using automated home cage monitoring system (Urban et al., 2011). However, these data provide information on inactivity, which does not necessarily represent sleep/rest duration. Sleep patterns recorded via electroencephalography and electromyography showed its impairment in CCI and arthritic model in rats (Landis et al., 1988; Monassi et al., 2003; Tokunaga et al., 2007; Guevara-López et al., 2009). Interestingly, we observed an increase in huddling behavior in both light and dark phases, possibly as a manifestation of a social-coping mechanism. In humans, it has been reported that the presence of others, particularly when they provide social support, can lead to a reduction of pain perception and anxiety (Mulej Bratec et al., 2020; Piedimonte et al., 2021). To our knowledge, our study is the first demonstration of an increase in huddling behavior in an animal model of chronic neuropathic pain. In rodents, D’Amato and Pavone reported a link between huddling among siblings and analgesic effect; their data showed that huddling between naïve/non-injured sibling mice led to an increase in nociceptive thresholds (D’Amato and Pavone, 1996). Research on huddling behavior has been widening beyond the field of evolutionary biology, where it was primarily associated with thermoregulation, to psychology and psychiatry and its use to evaluate social withdrawal in animal models of disorders such as autism, schizophrenia and post-traumatic stress disorder (Naskar et al., 2019; Zhao et al., 2023). Huddling is one of the most important displays of social interaction in rodents for adaptation to the environment (Zhao et al., 2023). Neurophysiological and neuroimaging evidence suggests that social, affective touch represents a distinct tactile experience, primarily associated with social interactions and relationships. Social touch is believed to help regulate physiological responses to acute stress and may mitigate maladaptive stress reactions. As such, it is considered an important stress-coping mechanism, contributing to emotional and physical well-being by buffering the negative effects of stress (Morrison, 2016). Pain is widely regarded as a stressful event due to its activation of both physiological and psychological stress responses (Flood and Clark, 2016; Pagé et al., 2021). In chronic pain, prolonged activation of stress pathways may contribute to increased stress behaviors. This prolonged stress response could explain the observed increase in huddling, which may serve as a marker of pain-related behavior, reflecting the body’s attempt to cope with pain-induced stress through social and physical proximity. Moreover, it was found that social animals, such as humans and rodents, consider the company of others as a strong signal of safety and such reliance on others for emotional regulation is an adaptive trait (Zhao et al., 2023). Considering sex differences, our data reveal increased huddling in females during the light phase. Curiously, it has been reported that exogenous testosterone administration induced huddling behavior (Zhao et al., 2023); the same study also demonstrated a positive correlation between huddling time and serum testosterone. On the other hand, we did not find SNI-induced differences in play fighting or social interaction. Khosravi and colleagues investigating social and aggressive behavior in pain models demonstrated deficits in social novelty preference in the three-chamber test, and increased aggressive behavior in resident/intruder test, while animals’ sociability in the three-chamber test was not impaired (Khosravi et al., 2021). However, it is important to note that our results come from undisturbed, home-cage observations while the majority of the studies used commercially available tests that produce novelty to the animal which can impact the results. Regarding play fighting, our data corroborate previous evidence that males spent more time play fighting compared to females (Pellis and Pellis, 1990; Gatewood et al., 2006).

Although we did not observe SNI-induced effects on grooming, which is in line with previous studies (Benbouzid et al., 2008; Silva-Cardoso et al., 2021), we found increased grooming in males compared to females during the dark phase. Previous work in rats demonstrated increased grooming in males exposed to a novel juvenile conspecific compared to females with the same condition (Thor et al., 1988). Another study with a 23 h observation of 2.5 months old mice did not observe any sex difference in grooming duration but 21.5 months old female mice showed increased grooming between 3 p.m. and 7 p.m. (light phase) compared to male mice (Tran et al., 2021).

## 5 Conclusion

Sensory disturbances like allodynia are just a small part of the symptom set of patients with chronic pain. For instance, in a cross-sectional study with 1,236 participants, only 20% of patients with neuropathic pain reported mechanical allodynia (Clark, 2016). Therefore, to increase the translational power of animal models of chronic pain, pain-related readouts have to be meaningful and adequate to the pathological characteristics of the modeled disease (Clark, 2016; González-Cano et al., 2020). Identifying more informative behaviors related to ongoing pain is of particular importance for improving the validity of these models. In our study on spontaneous home-cage behaviors we demonstrated that the SNI model of chronic neuropathic pain is associated with alterations in such behaviors, with differences between sexes. These findings open a new perspective into pain evaluation and support the contention that considering spontaneous behaviors may be valuable when assessing pain-like behavior in rodents. However, further studies (e.g., attenuation with analgesics) are required to validate these behavioral alterations specifically as pain outcome measures.

## Data Availability

The raw data supporting the conclusions of this article will be made available by the authors upon request.
