# Supplementary material for: Characterization of alterations in spontaneous behaviors in a rat model of neuropathic pain - new outcome measures for pain evaluation?
Source: Front Behav Neurosci. 2025 Jun 6;19:1550476. doi: 10.3389/fnbeh.2025.1550476 (PMC12179769; doi:10.3389/fnbeh.2025.1550476)
Supplement: Supplementary file 1 [file Table_1.docx]

Supplementary Table 1. Surgery and sex effects on home-cage behaviours during the dark phase analysed by Two-way ANOVA.

| Behaviour | Factor | df | F | p |
| --- | --- | --- | --- | --- |
| Huddling | **Surgery**  Sex  Surgery*Sex | 1, 31  1, 31  1, 31 | 4.406  0.544  1.777 | **0.044**  0.466  0.192 |
| Exploring | Surgery  Sex  Surgery*Sex | 1, 31  1, 31  1, 31 | 3.967  0.001  1.979 | 0.055  0.972  0.169 |
| Play fighting | Surgery  **Sex**  Surgery*Sex | 1, 31  1, 31  1, 31 | 1.795  54.219  0.051 | 0.190  **˂ 0.001**  0.823 |
| Self-grooming | Surgery  **Sex**  Surgery*Sex | 1, 31  1, 31  1, 31 | 0.051  8.190  0.004 | 0.822  **0.007**  0.947 |
| Social interaction | Surgery  Sex  Surgery*Sex | 1, 31  1, 31  1, 31 | 2.721  2.562  1.155 | 0.109  0.120  0.291 |
| Central cage absence | Surgery  Sex  Surgery*Sex | 1, 31  1, 31  1, 31 | 3.025  1.149  0.005 | 0.092  0.292  0.942 |

Supplementary Table 2. Surgery and sex effects on home-cage behaviours during the dark phase analysed by Kruskal-Wallis test.

| Behaviour | Comparison | H | p | Mann-Whitney U | p-value | Bonferroni-Holm  corrected p-value |
| --- | --- | --- | --- | --- | --- | --- |
| Following/chasing |  | 12.84 | **0.005** |  |  |  |
|  | SNI male vs SNI female |  |  | 15 | **0.0005** | 0.0125 |
| Sleep/rest alone |  | 1.120 | 0.772 |  |  |  |

Supplementary Table 3. Surgery and sex effects on home-cage behaviours during the light phase analysed by Two-way ANOVA.

| Behaviour | Factor | df | F | p | Post Hoc t | p_tukey_ |
| --- | --- | --- | --- | --- | --- | --- |
| Huddling | **Surgery**  **Sex**  **Surgery*Sex**  **SNI male vs. SNI female**  **Sham male vs. sham female**  **SNI male vs. sham male** | 1, 31  1, 31  1, 31 | 9.623  52.506  4.399 | **0.004**  **˂ 0.001**  **0.044** | 4.394  5.765  3.649 | **˂ 0.001**  **˂ 0.001**  **0.005** |
| Central cage absence | **Surgery**  **Sex**  Surgery*Sex | 1, 31  1, 31  1, 31 | 6.460  0.053  0.053 | **0.016**  0.819  0.819 |  |  |

Supplementary Table 4. Surgery and sex effects on home-cage behaviours during the light phase analysed by Kruskal-Wallis test.

| Behaviour | Comparison | H | p | Mann-Whitney U | p-value | Bonferroni-Holm  corrected p-value |
| --- | --- | --- | --- | --- | --- | --- |
| Exploring | SNI male vs SNI female  SNI male vs Sham male | 9.162 | **0.0272** | 28  11 | 0.0173  0.0264 | 0.0125  0.0167 |
| Self-grooming | SNI male vs SNI female  SNI male vs Sham male | 17.48 | **0.0006** | 21  5.5 | **0.004**  **0.0032** | 0.0167  0.0125 |
| Sleep/rest alone |  | 2.081 | **0.556** |  |  |  |

Supplementary Table 5. Two-way repeated measures ANOVA significant results of within subject effect of time point x surgery x sex interaction.

| Behaviour | Phase | df | F | p |
| --- | --- | --- | --- | --- |
| Huddling | Dark | 2.569,79.645 | 4.793 | 0.006 |
| Exploring | Dark | 4,124 | 11.007 | ˂ 0.001 |
| Play fighting | Dark | 4,124 | 5.198 | ˂ 0.001 |
| Following/chasing | Dark | 4,124 | 2.837 | 0.027 |
| Huddling | Light | 4,124 | 4.979 | ˂ 0.001 |
| Central cage absence | Light | 4,124 | 3.548 | 0.009 |

Supplementary Table 6. Tukey *post hoc* significant between group comparisons of data in Table 5.

| Behaviour | Phase | Comparison | Time point | t | p |
| --- | --- | --- | --- | --- | --- |
| Huddling | Dark | SNI M vs Sham M | POD7 | 4.230 | 0.006 |
|  | Dark | SNI M vs SNI F | POD7 | -3.823 | 0.025 |
|  | Dark | SNI M vs SNI F | POD21 | 3.721 | 0.035 |
|  | Dark | SNI F vs Sham F | POD28 | 4.828 | ˂ 0.001 |
|  | Dark | SNI M vs SNI F | POD28 | 5.010 | ˂ 0.001 |
|  | Light | SNI M vs Sham M | POD7 | 5.014 | ˂ 0.001 |
|  | Light | Sham M vs Sham F | POD7 | 4.130 | 0.010 |
|  | Light | SNI M vs SNI F | POD21 | 5.572 | ˂ 0.001 |
|  | Light | Sham M vs Sham F | POD21 | 5.468 | ˂ 0.001 |
|  | Light | SNI M vs SNI F | POD28 | 3.840 | 0.026 |
| Exploring | Dark | SNI F vs Sham F | POD7 | -3.680 | 0.041 |
|  | Dark | SNI F vs Sham F | POD14 | 3.868 | 0.023 |
|  | Dark | SNI F vs Sham F | POD28 | -4.565 | 0.002 |
| Play fighting | Dark | SNI M vs Sham M | POD14 | -4.145 | 0.008 |
|  | Dark | Sham M vs Sham F | POD14 | -5.339 | ˂ 0.001 |
|  | Dark | SNI M vs Sham M | POD21 | 4.844 | ˂ 0.001 |
|  | Dark | SNI M vs SNI F | POD21 | -6.087 | ˂ 0.001 |
| Following/chasing | Dark | SNI M vs SNI F | POD 21 | -6.850 | ˂ 0.001 |
| Central cage absence | Light | SNI M vs Sham M | POD7 | -4.168 | 0.010 |
|  | Light | SNI M vs SNI F | POD21 | -4.529 | 0.003 |
|  | Light | Sham M vs Sham F | POD21 | -4.141 | 0.011 |

*M-Male; F-Female

Supplementary Table 7. Tukey *post hoc* significant within group comparisons of data in Table 5.

| Behaviour | Phase | Sex | Surgery | Comparison | t | p |
| --- | --- | --- | --- | --- | --- | --- |
| Huddling | Dark | Male | SNI | POD7 vs POD14 | 4.072 | 0.012 |
|  | Dark | Male | SNI | POD7 vs POD21 | 5.628 | ˂ 0.001 |
|  | Dark | Male | SNI | POD7 vs POD28 | 5.078 | ˂ 0.001 |
|  | Dark | Female | SNI | POD7 vs POD 28 | -3.712 | 0.038 |
|  | Dark | Fenale | SNI | POD14 vs POD28 | -5.347 | ˂ 0.001 |
|  | Light | Male | SNI | POD7 vs POD21 | 5.632 | ˂ 0.001 |
|  | Light | Male | SNI | POD7 vs POD28 | 4.546 | 0.002 |
|  | Light | Male | SNI | POD14 vs POD21 | 4.433 | 0.003 |
| Exploring | Dark | Female | SNI | POD7 vs POD14 | -3.783 | 0.030 |
|  | Dark | Female | SNI | POD14 vs POD21 | 4.569 | 0.002 |
|  | Dark | Female | SNI | POD14 vs POD28 | 9.927 | 0.019 |
|  | Dark | Female | Sham | POD7 vs POD14 | 4.712 | 0.001 |
|  | Dark | Female | Sham | POD14 vs POD28 | -5.477 | ˂ 0.001 |
| Play fighting | Dark | Male | SNI | POD7 vs POD21 | -4.819 | ˂ 0.001 |
|  | Dark | Male | Sham | POD14 vs POD21 | 4.887 | ˂ 0.001 |
| Following/chasing | Dark | Male | SNI | POD7 vs POD21 | -6.000 | ˂ 0.001 |
|  | Dark | Male | SNI | POD14 vs POD21 | -6.338 | ˂ 0.001 |
|  | Dark | Male | SNI | POD21 vs POD28 | 6.796 | ˂ 0.001 |
| Social interaction | Dark | Male | SNI | POD14 vs POD21 | -3.655 | 0.045 |
|  | Dark | Female | SNI | POD14 vs POD21 | 3.830 | 0.026 |
